# Erythropoietin Modification Enhances the Protection of Mesenchymal Stem Cells on Diabetic Rat-Derived Schwann Cells: Implications for Diabetic Neuropathy

**DOI:** 10.1155/2017/6352858

**Published:** 2017-02-19

**Authors:** Shuyun Zhang, Baolin Shi

**Affiliations:** Department of Neurology, Weifang People's Hospital, Guangwen Street 151, Weifang 261041, China

## Abstract

Diabetes-triggered apoptosis of Schwann cells (SC) contributes to the degradation of diabetic peripheral neuropathy (DNP). In recent years, mesenchymal stem cells (MSC) were applied to DPN repair and it was demonstrated that paracrine secretion played a key role in neuroprotection exerted by MSC. Erythropoietin (EPO) is a potent cytokine capable of reducing apoptosis of SC. However, the expression of EPO in MSC is limited. In this study, we hypothesized that overexpression of EPO in MSC (EPO-MSC) may significantly improve their neuroprotective potentials. The EPO overexpression in MSC was achieved by lentivirus transduction. SC derived from the periphery nerve of diabetic rats were cocultured with MSC or EPO-MSC in normal or high glucose culture condition, respectively. In normal glucose culture condition, the overexpression of EPO in MSC promoted the MSC-induced restoration of SC from diabetic rats, including increases in GSH level and cell viability, decrease in TUNEL apoptosis, upregulation of antiapoptotic proteins, p-Akt, and Bcl-2, and downregulation of proapoptotic proteins, cleaved caspase-3, and Bax. The subsequent results in high glucose culture condition showed similar promotions achieved by EPO-MSC. Thus, it could be concluded that EPO-MSC possessed a potent potential in hampering apoptosis of SC, and the suppression was probably attributed to attenuating oxidative stress and regulating apoptosis related protein factors.

## 1. Introduction

Diabetic peripheral neuropathy (DPN) is the most common, but least recognized and understood long-term complication of diabetes [1], which is the main risk for foot ulceration and eventual limb amputation. Schwann cells (SC), known as the peripheral myelin-forming cells, play a key role in maintaining nerve integrity during nerve injury [1–3]. SC are defenseless to hyperglycemia due to glucose uptake via the insulin-independent absorption way [4, 5]. The accumulated hyperglycemic toxicity would disorder the production of reactive oxygen species (ROS) and then impair antioxidant defense, ultimately resulting in apoptosis [3, 6]. High glucose-induced apoptosis of SC is highly linked to the pathogenesis of DPN [7], suggesting a potential target for therapeutic strategies against DPN [3].

Currently, there is a wide interest in using cell transplantation therapies, because some immature cells, such stem cells, could sustain the production of various trophic factors in vivo [8, 9]. Mesenchymal stem cells (MSC), one of widely used stem cells for their abundant autologous availability and delivery via an allogeneic fashion, not only possess multipotent differentiation properties [10, 11] but also are capable of secreting several cytokines and growth factors [12], which are of great potential value to restore nerve functions. Additionally, with developing technologies of cell culture and genetic engineering [13, 14], the therapeutic effects of MSC could be further promoted via integrating other well-established methods in vivo [15, 16] and in clinic [17], overcoming the serious side effects caused by clinical practice of monotherapy [18]. A variety of recent studies confirmed that MSC can repair DPN via differentiation into SC-like cells [19–21]. Nevertheless, scarce studies are focused on the paracrine secretion function of MSC for DPN therapies, not to mention a combined strategy with MSC.

Erythropoietin (EPO), primitively known for their hematopoietic effects, has been evidenced to possess neuroprotective function in neurodegenerative diseases recently [22]. Apart from erythropoiesis, EPO are capable of maintaining neural integrity [23, 24], through protecting against neuronal damage or promoting postinjury restoration [24, 25]. A current study has demonstrated that targeting dorsal root ganglion modified with EPO gene can suppress the progression of DPN [26]. Meanwhile, despite being not well-proven mechanism, EPO is revealed to be able to hamper the apoptosis of SC induced by glucose-mediated oxidative stress [27].

Based on the feasibility of EPO to repair DPN though enhancing SC survival, we herein introduced EPO gene into MSC through lentivirus transduction, for the purpose of promoting therapeutic effects of MSC. The effects were further confirmed by both indirect and direct culture methods with transwell culture system. In addition, we preliminarily studied the underlying mechanism of enhanced restoration of SC with EPO overexpression MSC enhanced, through the assessment of the level of antioxidant and the expression of apoptosis related proteins.

## 2. Materials and Methods

### 2.1. Ethical Approval

In this study, rats were raised and handled strictly according to the National Institutes of Health Guidelines on the Use of Laboratory Animals. Also, the related experimental protocols were careful designed and the approvals of the Committee on the Ethics of Animal Care and Use of Chinese People's Liberation Army General Hospital guaranteed the normalization of our study. We used our best effort which was made in reducing the sufferings of animal. Anaesthetization was performed using intraperitoneal injection of pentobarbital sodium in each surgery.

### 2.2. MSC Isolation and Culture

The MSC were isolated from the inguinal adipose tissue of rats (2-week-old Sprague-Dawley rats, Charles River Laboratories, Beijing, Co. Ltd.) and cultured according to the previous method [28]. Typically, the inguinal hair of rats was cut off, followed by 5-minute sterilization with 75% ethanol. Then, the adipose tissue isolation surgery was performed under sterile condition. The obtained adipose tissue was washed with phosphate buffer saline (PBS), until the contaminated debris and red blood cells were not observed on the tissue and in the washing liquid. The clean tissue underwent digestion for 30 min (0.1% collagenase I from Sigma and 0.05% trypsin in serum-free *α*MEM). Then, equal volume of *α*MEM (Gibco) containing 10% FBS (Hyclone) was supplemented to cease digestion. The mixture was filtered through 80 *μ*m mesh. The cell retentates were further placed on tissue culture dishes and incubated in *α*MEM (Gibco) with 10% FBS (Hyclone) at 37°C and 5% CO_2_. The adherent cells were selected as the first passage of MSC and these MSC were allowed to expand to the 5th passage.

### 2.3. Modification of EPO Expressing MSC

EPO lentiviral particles labeled by GFP (GenePharma, Shanghai, China) were applied to generate stable transfectants in MSC. The infected cells were screened with puromycin (1 *μ*g/mL) to finally attain MSC modified by EPO gene (EPO-MSC) and the transfections were performed, according to the manufacturer's protocols. Briefly, the MSC were seeded at 1 × 10^5^ cells/well in 6-well plates with *α*MEM for 24 h. After removing the culture media, the cells in each well were supplemented with 1 mL fresh medium and then infected with lentiviral particles concentrate [multiplicity of infection (MOI) 20 : 1] for 24 h at 37°C. Subsequently, after the removal of the transfection media, cells underwent washing treatment with PBS for several times. The acquired cells were then incubated with fresh culture medium. Two days later, the transfection activities of MSC were investigated by the expression of GFP through a fluorescent microscope. Those uninfected cells were removed by addition of puromycin (1 *μ*g/mL).

### 2.4. Establishment of Diabetic Model Rats

After an overnight fast, Sprague-Dawley rats (10 weeks old) underwent intraperitoneal injection of a citrate acid buffer solution (pH = 4.5) with streptozotocin (STZ, 65 mg/kg, Sigma) for the purpose of the inducement of diabetes, according to the previously described [29]. Age-matched rats were injected with citrate acid solution without STZ and served as the control group. Blood samples obtained from tail prick were applied for blood glucose measurement. The diabetic rats, which tolerated a blood glucose level higher than 250 mg/dL from 3 days to 8 weeks after STZ injection and possessed evident neuropathic hyperalgesia (mechanical threshold ≤ 5.0 g and thermal threshold ≤ 10 s, data not shown) at 8 weeks after STZ treatment, were selected for the following study.

### 2.5. SC Isolation and Culture

The isolation of SC was conducted according to previously reported method [27]. Typically, the STZ-induced diabetic model rats were anaesthetized using intraperitoneal injection of 4% pentobarbital sodium (1 mL/kg body weight). The bilateral sciatic nerves were isolated under sterile condition. After removing epineurium, the sciatic nerves were then cut with scissors into pieces. To attain dispersed cells, the small nerve fragments were digested with 1% collagenase IA (Sigma) and 0.5% trypsin (Sigma) in serum-free *α*MEM for 1 h at 37°C. Then, the DMEM/F-12 (Gibco) media containing 10% fetal bovine serum, 50 U/mL penicillin, 40 mg/mL streptomycin, and 0.3 mg/mL L-glutamine were added in order to cease digestion. The obtained mixtures were then filtrated through an 80 mesh sieve and cells were collected by centrifugation. Upon being resuspended by fresh culture media, the cells were seeds at 1.5 × 10^5^ cells/well into 24-well plates precoated with poly-L-lysine (0.1 mg/mL, Sigma). The high glucose condition was made by supplementing extra glucose (30 mM). The cells, which exhibited anti-S-100 immunofluorescence, were identified as SC.

### 2.6. MSC and SC Transwell Coculture

Indirect and direct coculture experiments of MSC and SC were both performed in our study. For indirect coculture experiments, the in vitro assay procedure to coculture MSC and SC in the transwell filter was conducted according to previously reported method [30]. Briefly, MSC were seeded at 1 × 10^5^ cells/well onto 24-well transwell permeable support (pore size: 0.4 *μ*m, Corning, NY, USA) and were incubated at 37°C, 5% CO_2_. After 12 h, SC at a density of 5 × 10^4^ cells/well were seeded into the bottom of 24-well plates. For direct coculture experiments, SC were seeded at 5 × 10^4^ cells/well onto 24-well plates. After attaching to plates, the cells were stained with DAPI. The excess dyes were washed out with PBS (pH 7.4). MSC were then seeded twice as many as SC onto the 24-well plates and cultivated in an incubator at 37°C, 5% CO_2_.

### 2.7. Evaluation of Cell Viability

Cell viability was detected with water-soluble tetrazolium salt-1 (WST-1), according to previously reported [27]. In brief, the culture media were incubated with 10 *μ*L of WST-1 reagent at 37°C, 5% CO_2_ for 4 h. The optical density (OD) value of each well in different groups was measured using a multifunctional enzyme-marking instrument. The cell viability was calculated by the following formula: cell viability (%) = (OD of experiment cells − OD of background)/(OD of control cells − OD of background) × 100%.

### 2.8. Cell Apoptosis Assay

Cell apoptosis was assessed using TUNEL Apoptosis Detection Kit (KeyGen, Nanjing, China), according to the manufacturer's protocol. The nuclei of cells were stained with DAPI. The numbers of TUNEL positive cells (TUNEL+) were counted in randomly selected 10 visual fields through fluorescent microscope. The level of cell apoptosis was calculated by dividing the amount of TUNEL+ cells by that of total cells.

### 2.9. Measurement of Total Glutathione Level

The level of GSH was analyzed with Total GSH Assay Kit (Beyotime), according to manufacturer's protocol. The total GSH level was expressed as nmol/mg protein.

### 2.10. Western Blotting

The lysis of cells exploited Laemmli Sample Buffer (Bio-Rad). After centrifugation at 4°C, proteins components were attained and protein levels were determined by BCA™ Protein Assay Kit (Thermo Scientific). Afterwards, 60 *μ*g of proteins was loaded for sodium dodecyl sulfate polyacrylamide gel electrophoresis (SDS-PAGE). The obtained discrete proteins were then transferred to nitrocellulose membranes; primary antibodies against p-Akt, Akt, caspase-3, Bax, or Bcl-2 were incubated over night at 4°C. Finally, the corresponding secondary antibodies marked with HRP were incubated for another 1 h at room temperature. Akt was used as the internal reference for p-Akt, while GAPDH served as internal reference for other three factors. The antibodies used in this study were all purchased from Cell Signal Technology.

### 2.11. Statistical Analysis

Data were showed as mean ± standard deviation (SD). *P* values < 0.05 were regarded as statistical significance. Analysis between the groups was performed by using one-way ANOVAs followed by Tukey's post hoc test for multiple pairwise examinations.

## 3. Results

### 3.1. Isolation and Cultivation of Peripheral Nerve Schwann Cells

**Figure 1:**
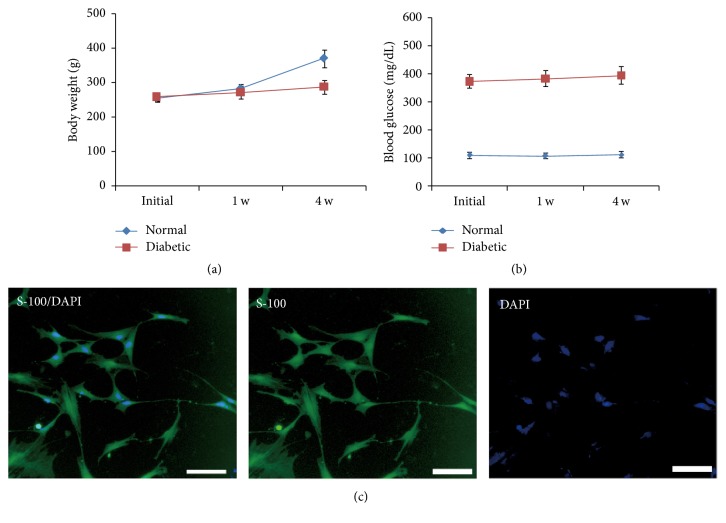
Establishment of diabetic model rats and isolation of SC. (a) The average body weight of rats in the control group increased time-dependently, while that in diabetes group almost kept constant. (b) After induction, the average concentration of blood glucose of rats in diabetes group was generally much higher than in control group. (c) SC isolated from sciatic nerve expressed S-100 protein.

Diabetic model rats were established by STZ induction. As shown in Figure 1, body weight of diabetic rats was lower than that of rats in control group after feeding for 4 weeks. The concentration of blood glucose in diabetic rats group (~400 mg/dL) was much higher than that in control group (~100 mg/dL). Subsequently, the SC derived from the bilateral sciatic peripheral nerve of diabetic model rats were isolated and cultivated. As shown in Figure 1(c), S-100 positive cells (green) were denoted to SC.

### 3.2. Protective Effects of EPO Overexpression MSC on Diabetic Rats SC through Indirect Coculture Method

**Figure 2:**
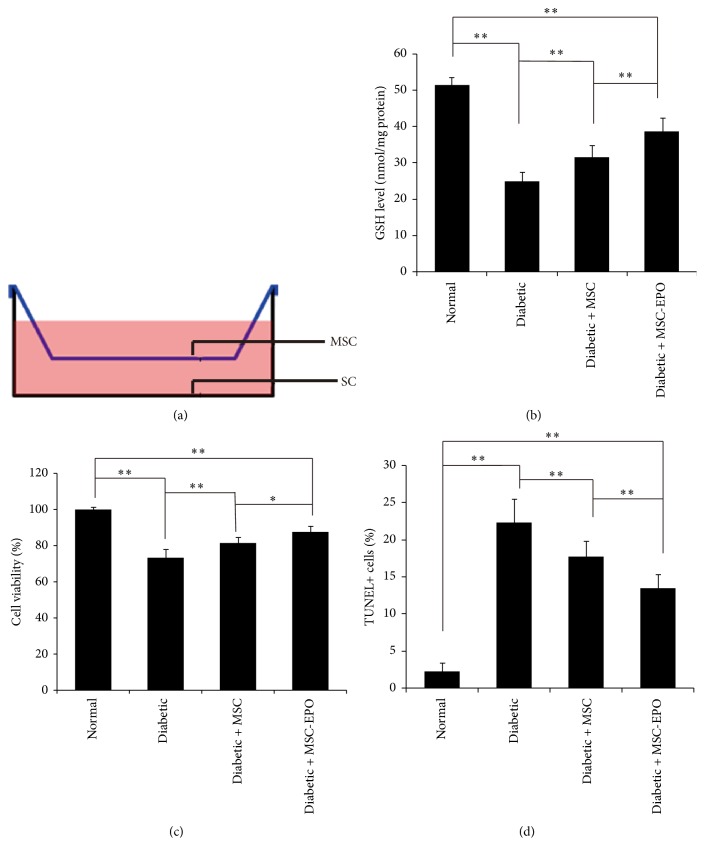
EPO overexpression MSC provided protection of SC derived from the periphery nerve of diabetic rat. (a) The schematic illustration of coculture of MSC and SC; (b) the cellular GSH level, (c) the cell viability, and (d) the cell apoptosis of SC in different groups; ^*∗*^*P* < 0.05; ^*∗∗*^*P* < 0.01.

As shown in Figure 2(a), we harnessed the indirect coculture of MSC and SC for the purpose of assessing the protective effects of MSC on SC. Compared with SC derived from normal rats (Figure 2(b)), the GSH level of SC from diabetic rats was remarkably lower, indicating a higher oxidative stress status in SC of diabetic rats. After coculture with MSC, the GSH level in SC of diabetic rats was significantly enhanced, demonstrating the protective effects of MSC on SC. Moreover, the cocultivation with MSC-EPO further significantly promoted the GSH level of SC derived from diabetic rats (versus MSC coculture group, *P* < 0.01). The results of GSH level manifested that the overexpression of EPO significantly strengthened the protection of MSC on SC. To better understand the influence of MSC on the living condition of SC, we next evaluated the cell viability and cell apoptosis (Figures 2(c) and 2(d)). Similarly, the coculture with MSC significantly enhanced the viability of SC and reduced the apoptotic level of SC, while the viability enhancement and the apoptotic decrease of SC were further extended by cocultivation with MSC-EPO.

**Figure 3:**
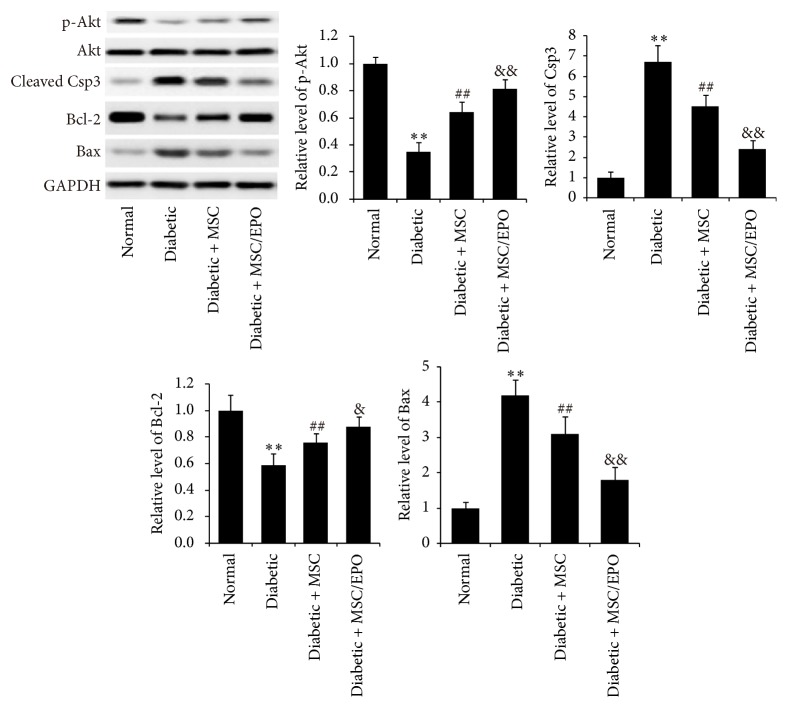
Expressions of apoptosis related proteins in SC indirect cocultivated with MSC-EPO. The protein expressions of their apoptosis related proteins, including p-Akt, cleaved caspase-3, Bcl-2, and Bax, were investigated by western blotting. ^*∗∗*^*P* < 0.01 compared with normal group; ^##^*P* < 0.01 compared with diabetic group; ^&&^*P* < 0.01 compared with diabetic + MSC group; ^&^*P* < 0.05 compared with diabetic + MSC group.

Furthermore, we employed western blotting technology to investigate the expression of cell apoptosis related protein. As shown in Figure 3, the western blotting results of apoptotic proteins complied with the former results of cell viability and TUNEL assay. The antiapoptosis related proteins of SC from diabetic rats, including p-Akt (*P* < 0.01) and Bcl-2 (*P* < 0.01), expressed significantly less abundantly, compared with those of normal rats, whereas the expressions of proapoptosis proteins, such as cleaved caspase-3 (*P* < 0.01) and Bax (*P* < 0.01), in SC of diabetic rats were highly significantly higher than those of control group. By contrast, after coculture with MSC, the expressions of p-Akt (*P* < 0.01) and Bcl-2 (*P* < 0.01) of SC were significantly increased, while the expressions of cleaved caspase-3 (*P* < 0.01) and Bax (*P* < 0.01) were significantly decreased. When SC from diabetic rats were cocultured with MSC/EPO, the expressions of p-Akt (*P* < 0.01), Bcl-2 (*P* < 0.05), cleaved caspase-3 (*P* < 0.01), and Bax (*P* < 0.01) offset significantly higher than those of MSC cocultured SC.

### 3.3. Protective Effects of EPO Overexpression MSC on Diabetic Rats SC through Direct Coculture Method

**Figure 4:**
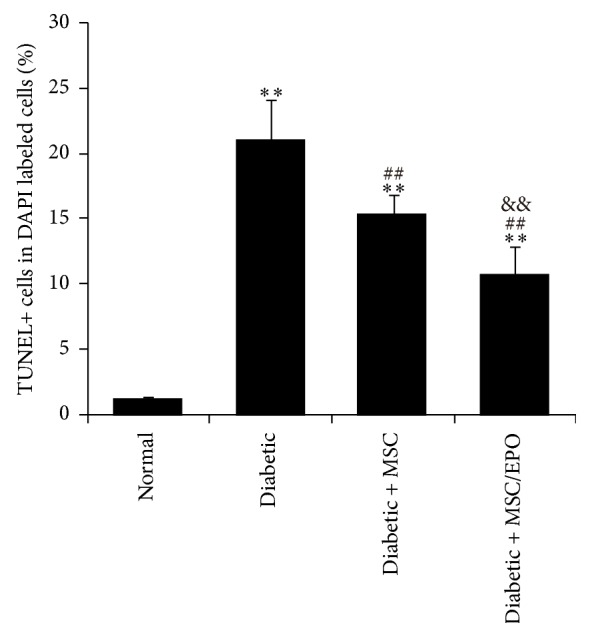
Apoptosis of SC directly cocultivated with MSC-EPO. The cellular apoptosis of SC in different groups was assessed by TUNEL staining. ^*∗∗*^*P* < 0.01 compared with normal group; ^##^*P* < 0.01 compared with diabetic group; ^&&^*P* < 0.01 compared with diabetic + MSC group.

To explore whether the coculture method would have an impact on the living condition of diabetic rats SC, we directly cocultivated SC and MSC on the 24-well plates. SC were prelabeled with DAPI to identify them from mixed cells. As illustrated by TUNEL staining results, after incubating with MSC for 24 h, apoptosis of SC significantly reduced, compared with that of diabetic SC without cocultivating with MSC. By contrast, the apoptotic level of SC cocultivated with MSC-EPO was further lowered, in comparison with that of MSC cocultured SC (*P* < 0.01), as showed in Figure 4.

### 3.4. Protective Effects of EPO Overexpression MSC on Diabetic Rats SC under High Glucose Condition through Indirect Coculture Method

**Figure 5:**
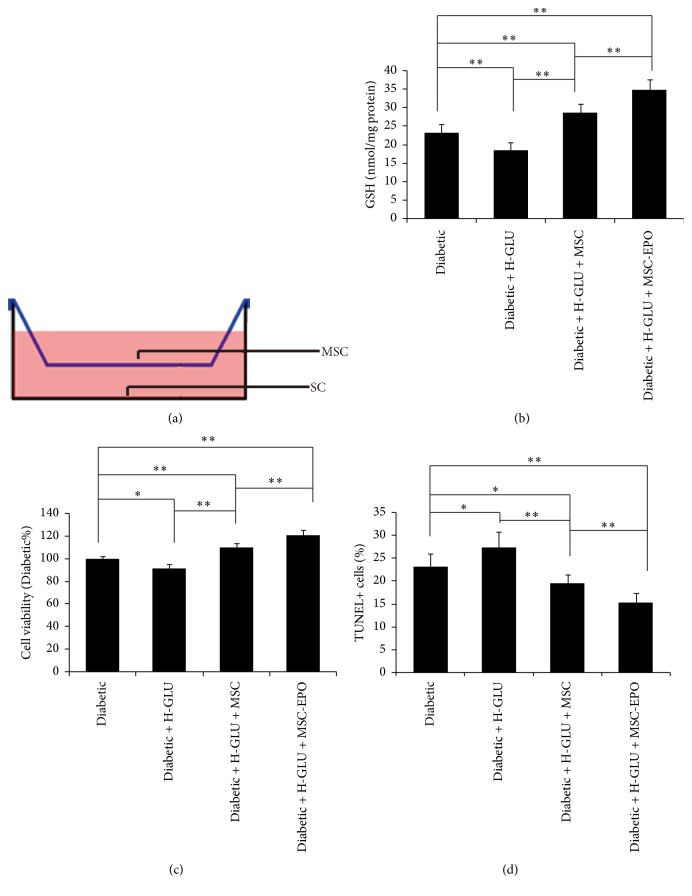
Under High glucose culture condition, indirect coculture with MSC-EPO provided protection of SC derived from diabetic rat. (a) The schematic illustration of indirect coculture of MSC and SC; (b) the cellular GSH level, (c) the cell viability, and (d) the cell apoptosis of SC in different groups; ^*∗*^*P* < 0.05; ^*∗∗*^*P* < 0.01.

We also employed high glucose (35.6 mM) culture condition to test the protection of SC by indirect coculture with MSC-EPO. As showed in Figure 5, compared with normal glucose (5.6 mM) culture condition, high glucose condition significantly decreased the cellular GSH level, reduced the cell viability, and increased the cellular apoptosis in SC, whereas those effects in SC induced by high glucose culture condition can be significantly attenuated by indirect cocultivating with MSC. Moreover, under high glucose culture condition, the GSH level in SC cocultured with MSC-EPO significantly increased, while cell viability and apoptosis of SC were further restored, compared with SC cocultured with MSC.

**Figure 6:**
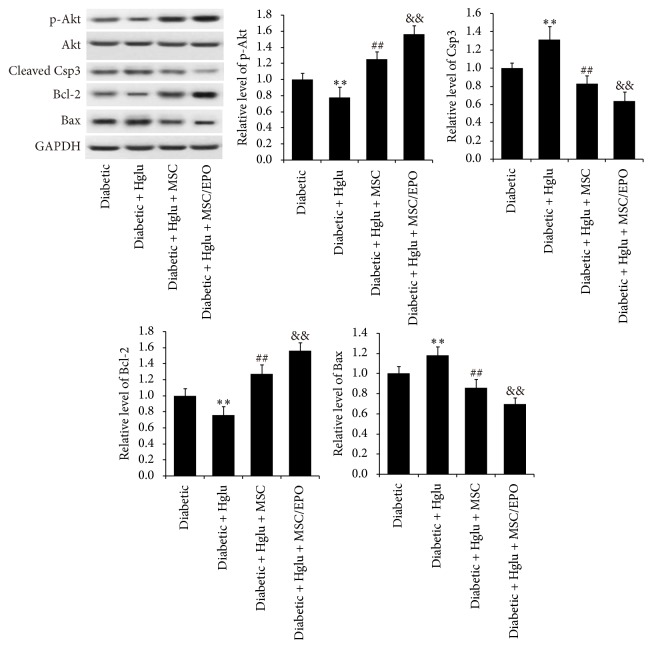
Expressions of apoptosis related proteins in SC indirect cocultivated with MSC-EPO under high glucose culture condition. The protein expressions of their apoptosis related proteins, including p-Akt, cleaved caspase-3, Bcl-2, and Bax, were investigated by western blotting. ^*∗∗*^*P* < 0.01 compared with normal group; ^##^*P* < 0.01 compared with diabetic group; ^&&^*P* < 0.01 compared with diabetic + MSC group.

Similarly, we investigated the expressions of apoptosis related proteins in SC under high glucose culture condition through western blotting. As showed in Figure 6, the results of protein expressions also conformed to the aforementioned cell viability and TUNEL assay. Under high glucose condition, the expressions of p-Akt (*P* < 0.01) and Bcl-2 (*P* < 0.01) in diabetic rats SC significantly attenuated, while there was more expression of cleaved caspase-3 (*P* < 0.01) and Bax (*P* < 0.01) than in control group. When cocultivated with MSC, SC would express more p-Akt (*P* < 0.01) and Bcl-2 (*P* < 0.01) and correspondingly less cleaved caspase-3 (*P* < 0.01) and Bax (*P* < 0.01). SC with a high glucose culture environment, which underwent coculture with MSC-EPO, had a significantly higher level expression of p-Akt (*P* < 0.01) and Bcl-2 (*P* < 0.01) as well as lower level expression of cleaved caspase-3 (*P* < 0.01) and Bax (*P* < 0.01), comparing with SC cocultured with MSC. These results of high glucose administration on diabetic rats SC further demonstrated the protective ability of MSC to SC, which could be considerably enhanced by EPO overexpression.

### 3.5. Protective Effects of EPO Overexpression MSC on Diabetic Rats SC Grown in High Glucose through Direct Coculture Method

**Figure 7:**
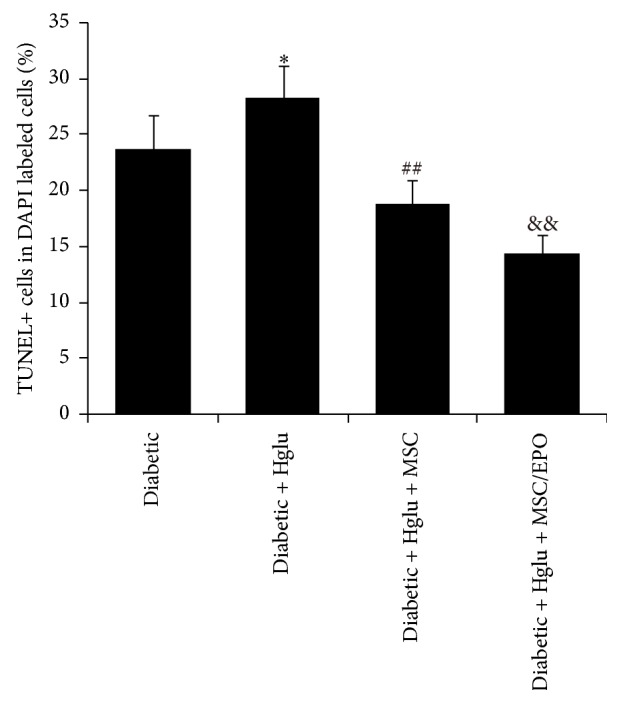
Apoptosis of SC directly cocultivated with MSC-EPO in high glucose culture condition. The cellular apoptosis of SC in different groups was assessed by TUNEL staining. ^*∗*^*P* < 0.05 compared with diabetic group; ^##^*P* < 0.01 compared with diabetic + high glucose group; ^&&^*P* < 0.01 compared with diabetic + high glucose + MSC group.

We also conducted direct cocultivation methods, to comprehensively verify the protective effects of MSC on SC. As showed in Figure 7, TUNEL staining results manifested that glucose-mediated apoptosis of MSC-treated SC significantly reduced. The inhibition of SC apoptosis by MSC could be strengthened by the overexpression of EPO. Together, these findings indicated that MSC overexpressed EPO in favor of promoting SC survival.

## 4. Discussion

The SC damage could lead to the reduction of cellular viability and ultimately apoptotic or autophagic death. Compared with normal SC, with the same culture condition, more of SC from diabetic rats suffered apoptosis. Their intracellular level of GSH was low, suggesting their vulnerability to cellular oxidants, such as ROS, and their viability also significantly descended. From a molecular level perspective, diabetic SC tended to express more of proapoptotic proteins and less of antiapoptotic ones. These results indicated the loss of cell integrity of diabetic SC, resulting in the decrease of their survival ability, which also were in accordance with previous reports [7, 27]. As shown in our data, the damage of SC induced by diabetes could be offset by the introduction of MSC. MSC were known not only to differentiate into specific cells but also to secrete a series of substances, like interleukins, chemokines, growth factors, colony-stimulating factors, and so forth [31–34]. Their paracrine roles are facilitated to improve their local or systematic microenvironment, which have been applied to a variety of studies of diseases. We observed that cocultivation with MSC significantly promoted the level of antioxidant and the expression of antiapoptotic proteins, while lowering the expression of apoptotic proteins, suggesting the protective role of MSC on diabetic SC.

The positive effects of MSC on SC are mainly ascribed to their paracrine secretion, which can be well established by coculture using transwell culture dishes. The factors and proteins produced and excreted by MSC can be transferred to SC and affect SC metabolism. Moreover, this indirect method favors the following biochemical analysis of SC, like GSH level assay, western blotting, and so forth. However, in the aspect of practical utility, MSC generally are direct injected to the problematic area, inevitably forming intercellular contacts. Intercellular contacts are capable of promoting repair capacity of radiation damage [35], deregulating DNA damage response pathway [36], and activating epidermal factors [37]. Despite difficulties in performing detailed cellular analysis, TUNEL staining is still applicative in apoptosis assessment of SC prelabeled with DAPI. Our data also further demonstrated that MSC could attenuate diabetes promoted apoptosis of SC, even by a directly mixed coculture way. Although plausible results, the therapeutic effects of diabetic SC with MSC are far from enough, compared with the normal SC.

Recently, EPO has been proved to be able to attenuate oxidative stress and apoptosis of SC from diabetic model rats [30], indicating that a better treatment can be achieved by upraising the EPO expression of MSC. The enhancement of EPO expression in MSC can be attained by several methods, like hypoxia-precondition [38], CoCl_2_ treatment [30], and so forth. Among those methods, gene transfection is superior due to less damage to cell and stable expression of target proteins. We thus employed lentivirus vector to modify MSC to overexpress EPO. Our data indicated that EPO overexpression promoted a nearly 2-fold increase in the therapeutic effects of MSC on diabetic SC. Also, the disorders of apoptosis related proteins expression were largely alleviated. Additionally, previous study has demonstrated the antioxidative stress properties of EPO on diabetic SC through elevating the total GSH level of SC, which conformed to our results. And our western blotting analysis of apoptotic proteins further indicated that EPO overexpression protected SC from apoptosis, associated with increased expression of antiapoptosis proteins, p-Akt, and Bcl-2 and decreased expression of proapoptosis proteins, cleaved caspase-3, and Bax [39].

Assessments of SC living status under high glucose culture condition are necessary, for better imitating real diabetic living conditions of SC. The producing of ROS could be a possible explanation for high glucose-induced SC dysfunction in both in vitro and in vivo studies [40, 41]. Our data that high glucose culture of SC is linked to total GSH level uplifting and cellular apoptosis increase also partially proved that. The changing in ROS level may open the permeability transition pores of mitochondria, a key source, and target of ROS, resulting in releasing apoptosis-activating proteins [42]. Antiapoptotic protein, Bcl-2, and proapoptotic protein, Bax, on the membrane outer layer of mitochondria are essential for cell survival. Apoptotic stimuli initiated Bax expression, inhibited Bcl-2 expression, and also triggered the release of CytoC, which ultimately activated the late-stage apoptotic protein, cleaved caspase-3 [43, 44]. In the present study, high glucose culture in SC upregulated the expression of Bax and cleaved caspase-3 and downregulated Bcl-2 expression, compared with normal glucose administration. The further investigations of the effects of EPO overexpression MSC on high glucose-induced detrimental effects were in accordance with the former results by normal glucose cultivation, confirming the synergistic promotion of EPO and MSC on diabetic SC.

Herein, the present study conducted a comprehensive assessment of EPO overexpression MSC on SC, combining with phytochemical and physiological studies. Conclusively, the overexpression of EPO promoted the therapeutic effects of MSC on diabetic neuropathy via inhibiting SC apoptosis.
